# Trustworthy intelligent rooms: integrating blockchain, federated learning, and data-centric AI for healthcare 4.0

**DOI:** 10.3389/fdgth.2026.1758304

**Published:** 2026-04-22

**Authors:** Ramesh Kumar Veerapaneni, Radhakrishnan Delhibabu

**Affiliations:** School of Computing Science and Engineering (SCOPE), Vellore Institute of Technology, Vellore, India

**Keywords:** ambient intelligence, blockchain, data-centric AI, federated learning, healthcare 4.0, trustworthy AI

## Abstract

**Introduction:**

Intelligent room systems are experiencing a surge in demand within the Healthcare 4.0 ecosystem. The integration of Federated Learning (FL) and Data-Centric AI has led to substantial enhancements in the predictive capabilities of machine learning models while maintaining data privacy. However, centralized aggregation in FL remains a single point of failure and is vulnerable to poisoning attacks.

**Methods:**

This paper presents a novel, privacy-preserving architecture for Ambient Intelligence (AmI) that integrates Distributed Ledger Technology (DLT).

**Results:**

We explicitly note that while DLT does not preemptively prevent the generation of poisoned gradients, it provides an immutable, cryptographically secure audit trail. This ensures the trustworthiness and traceability of model updates for post-hoc detection, strict accountability, and targeted model rollbacks.

**Discussion:**

By fusing Data-Centric AI for quality assurance with a Blockchain-enabled FL framework, we propose a scalable, low-cost solution for real-time patient monitoring in diverse economic settings.

## 1 Introduction

Medical advancements over recent decades have paradoxically increased the complexity of care. As life expectancy rises, patients increasingly present with multi-morbid conditions, requiring continuous and sophisticated monitoring. This demographic shift has intensified medical specialization, creating a significant disparity in care delivery. Rural hospitals, in particular, often lack the infrastructure to provide comprehensive, high-quality care comparable to urban centers. Existing systems typically by a shrinking workforce of skilled medical professionals, a trend that is particularly acute in developing Asian economies (1).

Financial constraints on healthcare institutions further complicate the management of complex diseases among aging populations. Traditional models of care, which rely heavily on direct physician-patient interaction, are becoming financially unsustainable. To maintain health standards without incurring prohibitive costs, advanced AI solutions are required. These solutions must essentially “clone” the vigilance of medical staff, alleviating their workload while ensuring efficient, real-time care delivery.

### 1.1 Limitations of current smart environments

While the concept of “Intelligent Rooms” or “Smart Hospitals” offers a potential solution, current implementations face significant hurdles. Numerous smart room solutions exist, but they often suffer from high deployment costs, limited functionality, and a critical lack of interoperability. Existing systems typically operate in silos, unable to seamlessly integrate with legacy infrastructure or adapt to diverse patient needs (2).

Furthermore, the reliance on centralized cloud processing for these systems introduces critical latency issues—unacceptable in scenarios like fall detection where milliseconds matter—and raises profound privacy concerns regarding the transmission of sensitive patient video data. The challenge, therefore, is not merely to build a smart room but to build one that is scalable, cost-effective, and inherently private.

### 1.2 The imperative for trustworthy and scalable AI

To address these challenges, this paper proposes a novel architecture leveraging Ambient Intelligence (AmI), IoT, Data-Centric AI, and Federated Learning (FL). However, standard Federated Learning architectures introduce a new vulnerability: the central aggregator. In a standard FL setup, if the central server is compromised, or if malicious nodes inject “poisoned” model updates, the global model’s integrity collapses.

This necessitates the integration of Distributed Ledger Technology (DLT). As detailed in recent surveys on secure computing (3), DLT transforms centralized databases into decentralized ledgers maintained by a network of participants. This necessitates the integration of Distributed Ledger Technology (DLT). As detailed in recent surveys on secure computing (3), DLT transforms centralized databases into decentralized ledgers maintained by a network of participants. By recording model updates on a blockchain, we eliminate the single point of failure inherent in traditional FL. It is important to emphasize that this DLT integration primarily provides an immutable audit trail for post-hoc attack detection and strict accountability, rather than preemptive attack prevention.

**Immutability:** Once a model update is recorded, it cannot be altered, providing an audit trail for medical AI decisions.
**Decentralization:** The system operates without a central authority, enhancing fault tolerance and security against localized attacks.
**Trust:** The use of cryptographic consensus mechanisms ensures that only valid, high-quality updates are integrated into the global patient monitoring model.

This paper presents a “Trustworthy AI” environment where Data-Centric methodologies ensure the quality of input data, FL ensures patient privacy, and DLT ensures the security of the learning process itself.

The remainder of this paper is structured as follows: Section 2 reviews related work in disease diagnosis support, patient monitoring, and Ambient Intelligence. Section 3 outlines the background technologies, including AmI, Federated Learning, and Data-Centric AI. Section 4 details the proposed solution, describing the general structure of AmI-based intelligent rooms, the FL architecture, and the Blockchain-enabled security layer. Section 5 presents a case study of the mobile application implementation. Section 6 presents several threats to validity that are acknowledged. Finally, Section 7 concludes the paper and discusses future research directions.

## 2 Related work

The evolution of Healthcare 4.0 has been driven by the convergence of the Internet of Medical Things (IoMT), Ambient Intelligence (AmI), and advanced machine learning techniques. This section reviews existing literature across three critical domains: Disease Diagnosis Support, Patient Monitoring Systems, and the emerging integration of Distributed Ledger Technology (DLT) for secure healthcare data management.

### 2.1 Disease diagnosis and clinical decision support

**Figure 1:**
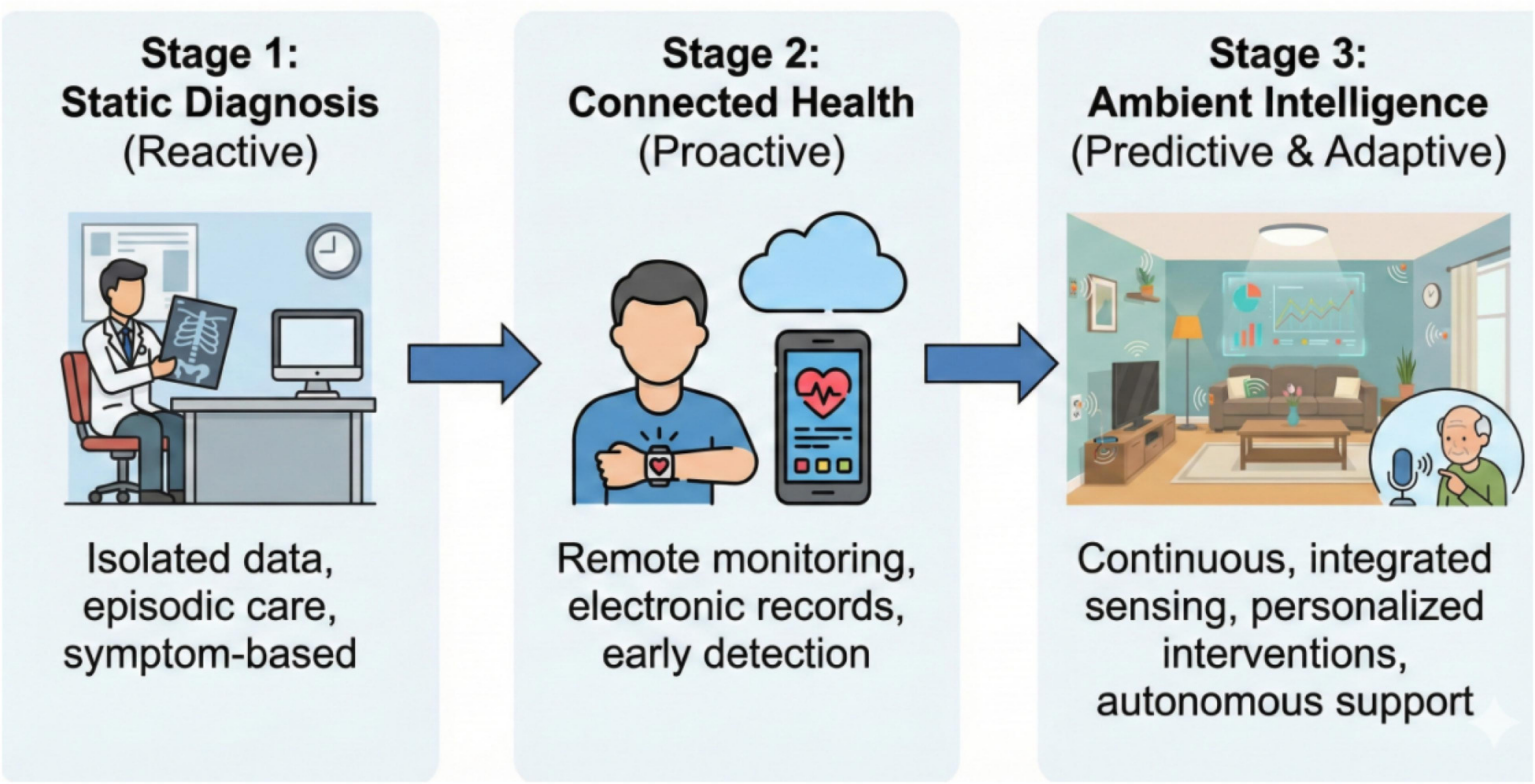
Evolution of healthcare systems from static diagnosis to ambient intelligence.

Clinical Decision Support Systems (CDSS) in Figure 1 have long been a cornerstone of digital health. These systems typically assist practitioners by offering differential diagnoses based on structured patient inputs.

A prominent example is **VisualDx** (4), a logic-based system designed to aid primary care practitioners in differential diagnosis. VisualDx utilizes a vast repository of over 100,000 medical images to rank potential conditions based on patient demographics and visual symptoms. While effective for educational purposes and static diagnosis, such systems operate as standalone software tools rather than integrated components of an intelligent environment. They lack real-time sensory input and cannot react to dynamic changes in a patient’s physical state within a hospital room.

### 2.2 Patient care and automated monitoring systems

To address the limitations of static diagnosis tools, recent research has focused on automated patient monitoring using Computer Vision and Deep Learning.

**Bedside monitoring:** Recent studies have proposed supervisory control systems for hospital beds using computer vision (5). These algorithms identify patient contours to track movements and automatically adjust the bed’s position to optimize comfort and safety. Some systems achieved high precision rates in identifying patient posture. Similarly, deep learning systems deployed in ICUs have been successful in detecting mobilization activities (6), such as a patient attempting to exit a bed, which is critical for preventing post-intensive care syndrome.

**Fall detection:** Specific solutions like the **Kepler Night Nurse (KNN)** utilize optical sensors and AI to detect falls in elderly care facilities. Unlike wearable sensors, which patients often forget to wear, KNN uses environmental sensors to generate alarms. Crucially, newer iterations include face-blurring features to address privacy concerns.

**Figure 2:**
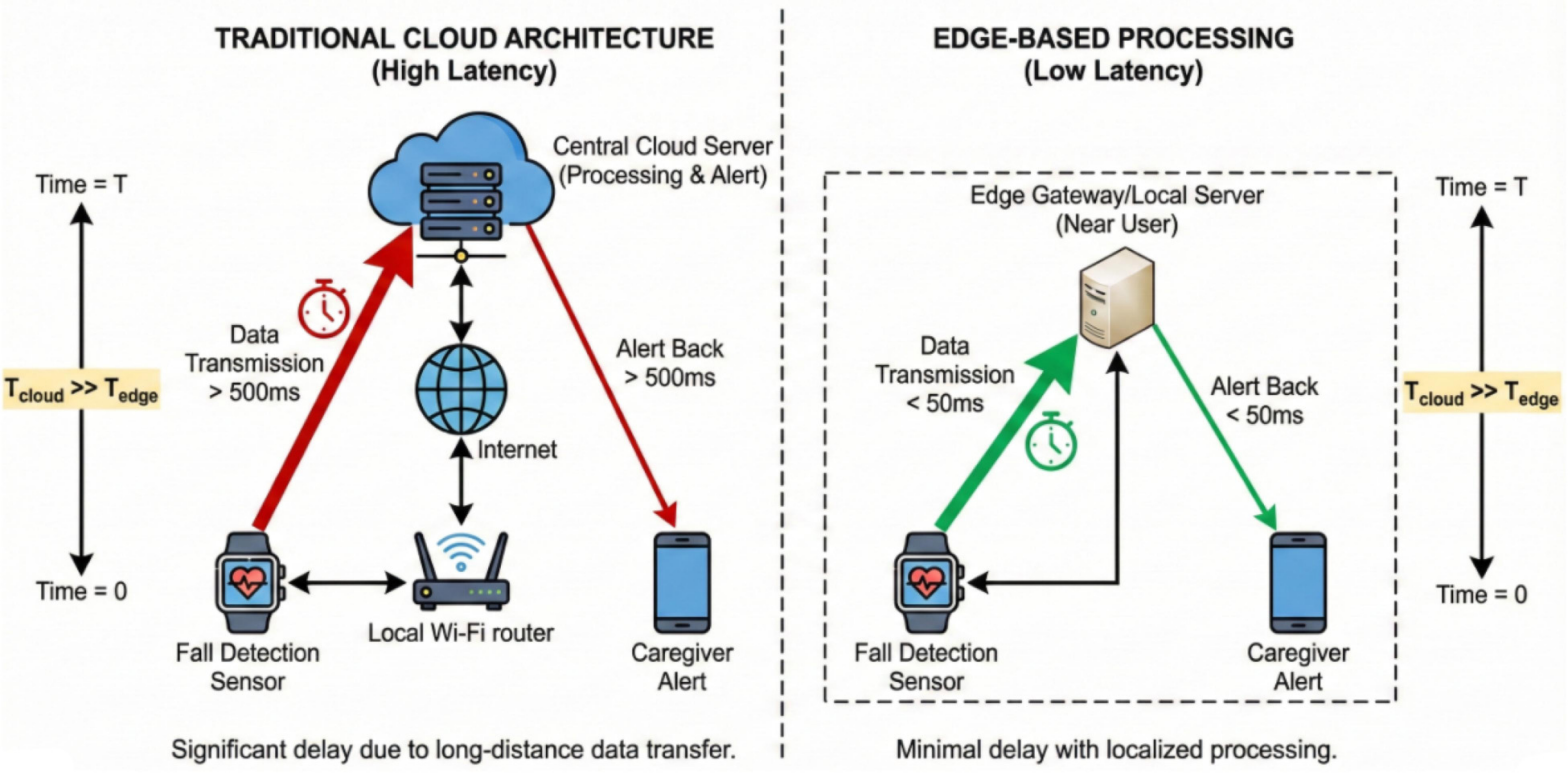
Latency comparison between traditional Cloud architectures and Edge-based processing for fall detection.

However, a recurring limitation in these systems is their architectural reliance on centralized processing. As shown in Figure 2, transferring high-bandwidth video data to a central cloud for inference introduces latency and creates a single point of failure—a critical vulnerability in life-critical monitoring scenarios.

### 2.3 Ambient intelligence (AmI) in healthcare

Ambient Intelligence represents the next leap in smart healthcare, creating environments that are sensitive, adaptive, and responsive to human presence.

Research has demonstrated smart environments that integrate wireless sensors to allow patients to control their room (lights, bed position) while simultaneously monitoring vital signs. These studies validated the utility of AmI in reducing the cognitive load on medical staff (13). However, most AmI implementations currently lack a mechanism for *continuous learning*. Once deployed, the AI models remain static unless manually updated, failing to adapt to specific patient behaviors over time. Table 1 summarizes the comparison of existing intelligent healthcare systems.

**Table 1:** Comparison of existing intelligent healthcare systems.

System type	Representative work	Key functionality	Limitations
Diagnosis support	VisualDx	Differential diagnosis via image matching.	Standalone software; no real-time environmental awareness.
ICU monitoring	Deep learning mobilization	Patient mobilization detection.	Centralized processing raises privacy risks; high bandwidth usage.
Fall detection	Kepler night nurse	Optical fall detection with privacy blurring.	Proprietary ecosystem; limited interoperability with other hospital systems.
Smart bed control	Computer vision control	Auto-adjustment based on patient contours.	Limited scope (bed only); does not utilize multi-modal data.

### 2.4 Trustworthy AI: the role of DLT and federated learning

The integration of Federated Learning (FL) and Distributed Ledger Technology (DLT) is emerging as a solution to the privacy and security bottlenecks identified above.

**Federated learning (FL):** FL enables the collaborative training of machine learning models without sharing raw patient data. This is particularly suited for medical image retrieval and classification, where data confidentiality is mandated by regulations like HIPAA or GDPR.

**Figure 3:**
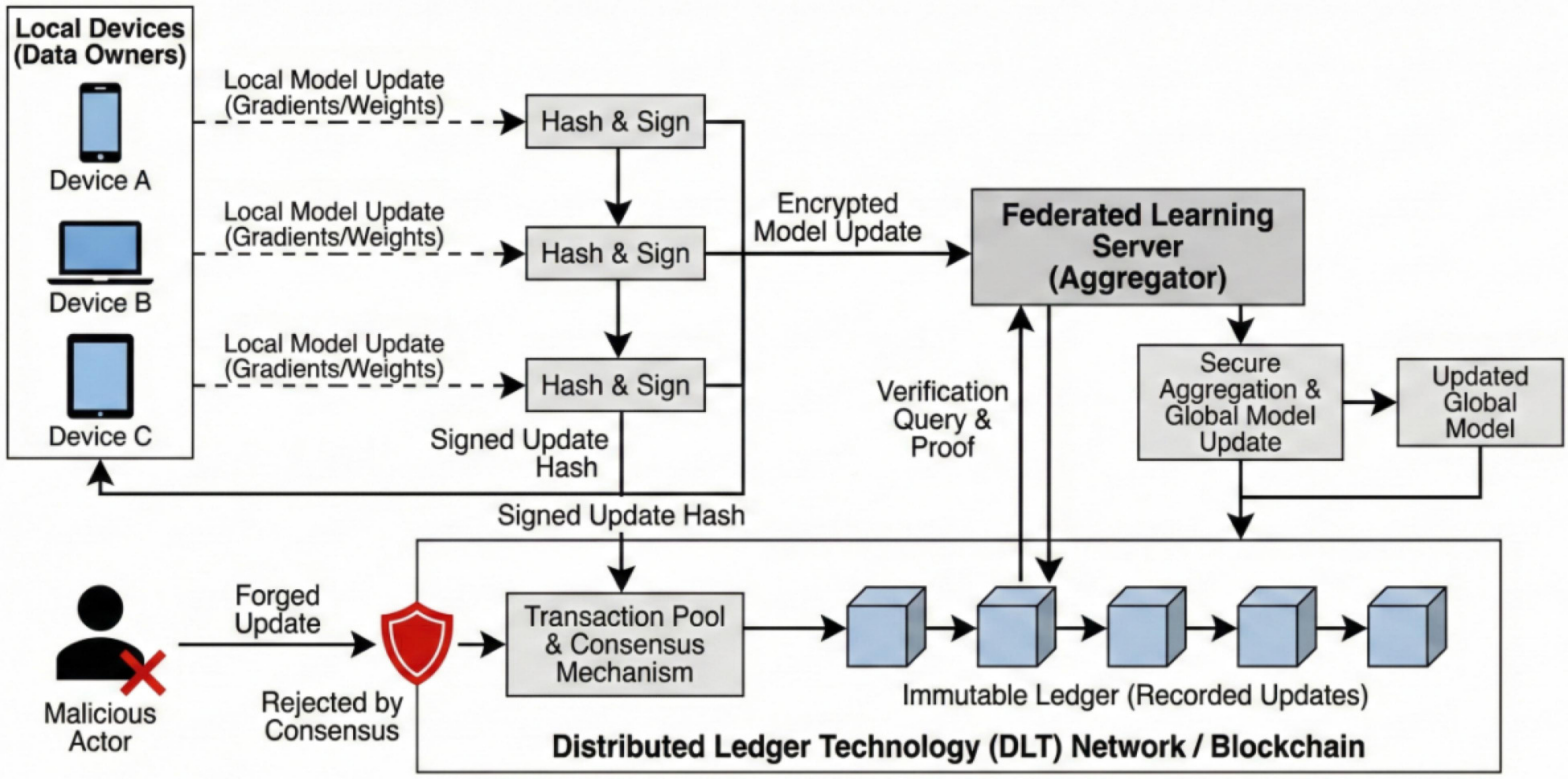
Integration of DLT for securing federated learning model updates.

**Distributed ledger technology (DLT):** While FL protects data privacy, it is vulnerable to “poisoning attacks,” where malicious nodes upload corrupt model updates. DLT offers a robust countermeasure. As detailed in recent surveys on secure computing, blockchain provides an immutable audit trail. By recording model updates on a ledger, the system ensures traceability and accountability. Figure 3 shows the integration of DLT for securing Federated Learning model updates.

Table 2 summarizes the architectural shift proposed in this paper compared to traditional approaches. While centralized systems offer simplicity, they fail in privacy and latency. Pure FL addresses privacy but lacks the trust layer essential for medical liability. Our proposed hybrid DLT-FL approach bridges this gap.

**Table 2:** Comparative analysis of privacy and security architectures.

Architecture	Data privacy	Latency	Trust & immutability
Centralized cloud AI	Low (Data sent to cloud)	High	Low (Opaque processing)
Edge computing	Medium (Local processing)	Low	Low (Local logs only)
Standard federated learning	High (Weights only)	Medium	Medium (Vulnerable to poisoning)
**Proposed DLT-enabled FL**	**High**	**Low**	**High (Immutable Ledger)**

Bold text indicate the optimal parameters or the superior performance metrics achieved by the proposed architecture.

Recent advancements have increasingly explored the intersection of IoT, blockchain, and federated learning to secure healthcare data. For instance, Barbaria et al. (7) proposed a combined Blockchain and IoT high-level architecture tailored for patient monitoring systems, emphasizing secure data acquisition. This was further expanded to distributed networks for patient information sharing (8), and more recently, applied to ensure strict compliance with HIPAA and GDPR mandates during secure data exchange in clinical research (9). Similarly, Chowdhury and Kudapa (10) highlighted the efficacy of Federated Learning models in facilitating privacy-preserving data sharing across healthcare institutions. Pushing the security paradigm further, Ravisankar and Maheswar (11) introduced SecureEdge-MedChain, integrating post-quantum blockchain with FL for the Internet of Medical Things (IoMT). While these studies establish the theoretical viability of blockchain and FL in healthcare, our work distinctively integrates these paradigms with a Data-Centric AI quality assurance loop tailored specifically for real-time Ambient Intelligence (AmI) environments.

In summary, while individual technologies for diagnosis, monitoring, and privacy exist, there is a lack of a unified framework that combines the real-time responsiveness of AmI with the security of DLT. This paper addresses this convergence, proposing a scalable solution suitable for resource-constrained Healthcare 4.0 environments.

## 3 Background technology

The proposed architecture for trustworthy intelligent rooms resides at the intersection of three distinct technological domains: Ambient Intelligence for sensing, Federated Learning for decentralized computation, and Data-Centric AI for quality assurance. Understanding the interplay between these technologies is crucial for addressing the challenges of privacy, latency, and trust in Healthcare 4.0.

### 3.1 Ambient intelligence (AmI) in healthcare ecosystems

**Figure 4:**
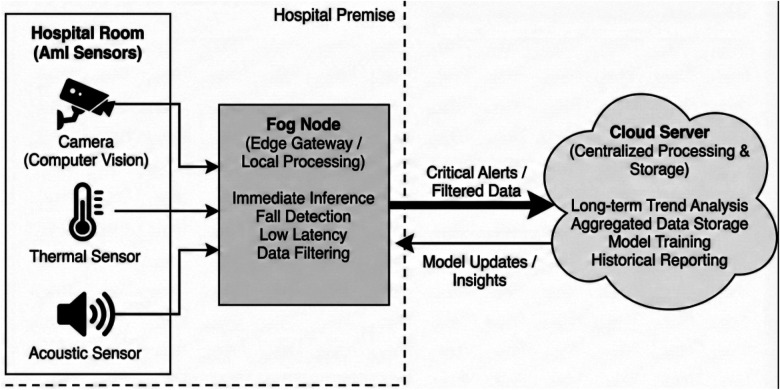
Hierarchical data processing in an AmI-enabled hospital room.

Ambient Intelligence refers to electronic environments that are sensitive and responsive to the presence of people. In a healthcare context, AmI transforms a passive hospital room into an active partner in patient care. Unlike traditional monitoring that relies on obtrusive wearable devices—which elderly patients often forget or refuse to wear—AmI utilizes environmental sensors embedded in the infrastructure. Figure 4 shows the hierarchical data processing in an AmI-enabled hospital room.

A robust AmI system in a hospital setting typically relies on multi-modal sensor fusion. This involves integrating disparate data streams to build a coherent model of patient behavior:

- **Computer vision sensors:** Optical cameras (often privacy-masked or using depth-only sensing) provide skeletal tracking for pose estimation, crucial for fall detection and mobility assessment.
- **Thermal imaging:** Used for non-contact temperature monitoring and detecting bed occupancy without revealing personally identifiable visual features.
- **Acoustic sensors:** Capable of detecting calls for help, sounds of distress, or specific acoustic patterns associated with a fall.

The data processing pipeline for AmI demands a hierarchical approach. Transmitting raw, high-definition video feeds from dozens of rooms to a central cloud is impractical due to bandwidth constraints and latency requirements. Therefore, modern AmI architectures utilize a Fog-Cloud continuum. Critical, time-sensitive inference (e.g., fall detection) occurs at the network edge (Fog nodes within the hospital), while long-term trend analysis occurs in the cloud.

### 3.2 Federated learning: collaborative training with privacy

Standard machine learning approaches require centralizing data from all sources into a single repository for training. In healthcare, this is often prohibited by strict regulations regarding patient data privacy and data sovereignty. Federated Learning (FL) addresses this paradigm shift by enabling collaborative model training without moving raw patient data from the local devices (edge nodes or hospital servers).

In a typical Federated Averaging (FedAvg) setup:

A central server initializes a global neural network model.
This model is broadcasted to participating client nodes (e.g., different hospitals or individual smart rooms).
Each client trains the model on its own local, private dataset for several epochs.
Instead of sharing data, clients share only their updated model weights (gradients) back to the central server.
The server aggregates (averages) these updates to create a new global model, which is then sent back to the clients for the next round.

**Figure 5:**
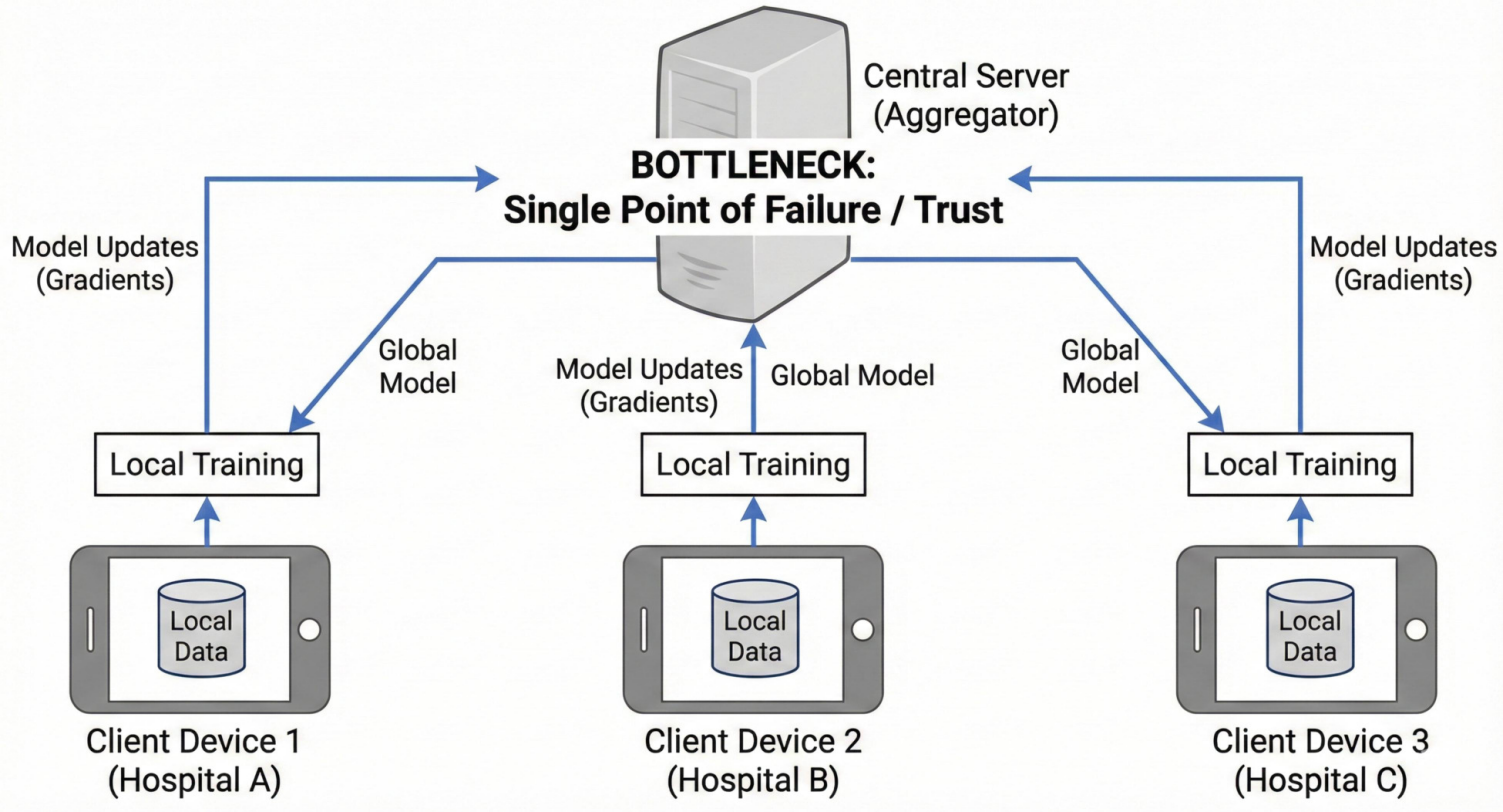
Standard federated learning and the central aggregator bottleneck.

Figure 5: While FL significantly enhances privacy, standard implementations introduce a critical vulnerability: reliance on a central aggregator. This central server constitutes a single point of failure. Furthermore, it requires implicit trust that the server will aggregate correctly and that participating clients are not malicious. A compromised client could engage in “model poisoning,” injecting malicious gradients designed to corrupt the global model’s performance on specific tasks (e.g., ensuring falls go undetected for specific patient demographics).

### 3.3 The shift to data-centric AI

Historically, AI development has been “model-centric,” focusing on iterating model architectures and hyperparameter tuning while treating the data as a fixed input. However, in real-world medical deployments, the primary bottleneck to performance is rarely the model architecture but the quality of the data.

Data-Centric AI flips this paradigm by treating code (the model) as fixed and focusing engineering efforts on systematically improving dataset quality (14, 15). In the context of intelligent rooms, medical data is inherently noisy:

- **Label noise:** Labels provided by overworked medical staff may be inconsistent. What one nurse classifies as a “near-fall,” another might dismiss.
- **Class imbalance:** Critical events like falls are rare compared to normal activities like sleeping or sitting. A model trained on raw data will be heavily biased toward normal classes.
- **Domain shift:** A model trained in a brightly lit room may fail in a dimly lit room at night.

A Data-Centric approach involves building automated pipelines for cleaning data, using generative techniques to augment rare classes, and implementing human-in-the-loop systems where ambiguous examples are flagged for expert review. This ensures that the local models in the federated network are trained on high-quality representations, preventing “garbage in, garbage out.”

### 3.4 Convergence towards trustworthy AI

While AmI provides the senses, FL provides the privacy, and Data-Centric AI provides quality, a gap remains in guaranteeing *trust*. Trustworthy AI in healthcare requires accountability and auditability. If an AI system fails to detect a critical event, medical liability requires a traceable log of why the model behaved that way.

Standard FL is opaque; there is no verifiable record of which client submitted which update, or if the aggregation was performed honestly. To bridge this gap, technologies that establish decentralized trust are required to govern the federated process, ensuring that the resulting environmental intelligence is not only smart but also verifiable and secure.

**Figure 6:**
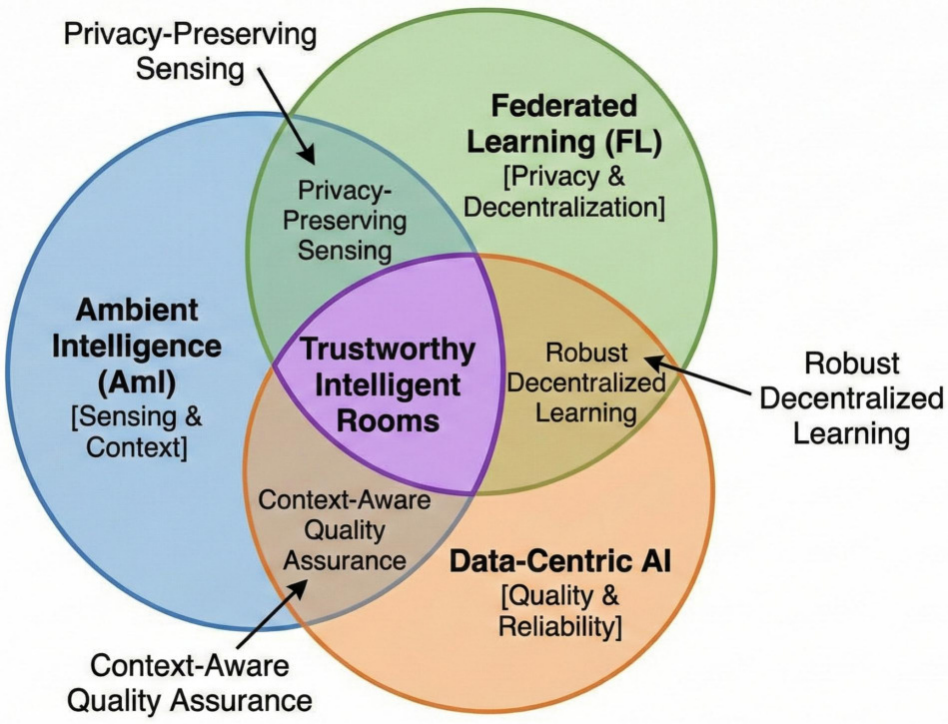
Convergence of technologies required for trustworthy intelligent rooms.

Figure 6 illustrates how these technologies converge to form the proposed system, and Table 3 compares standard approaches with the necessary requirements for trustworthy medical AI.

**Table 3:** Learning paradigms in healthcare context.

Feature	Centralized learning	Standard federated learning	Trustworthy federated learning (Goal)
Data privacy	Low (Raw data leaves premise)	High (Data stays local)	High (Data stays local)
Single point of failure	Yes (Central Data Server)	Yes (Central Aggregator)	No (Decentralized Aggregation)
Audit trail/immutability	Low (Database logs, mutable)	Low (Ephemeral updates)	High (Immutable ledger required)
Resilience to poisoning	Medium (Easier to audit central data)	Low (Hard to detect malicious local updates)	High (Verifiable updates required)

## 4 Proposed solution: the trustworthy ami architecture

**Figure 7:**
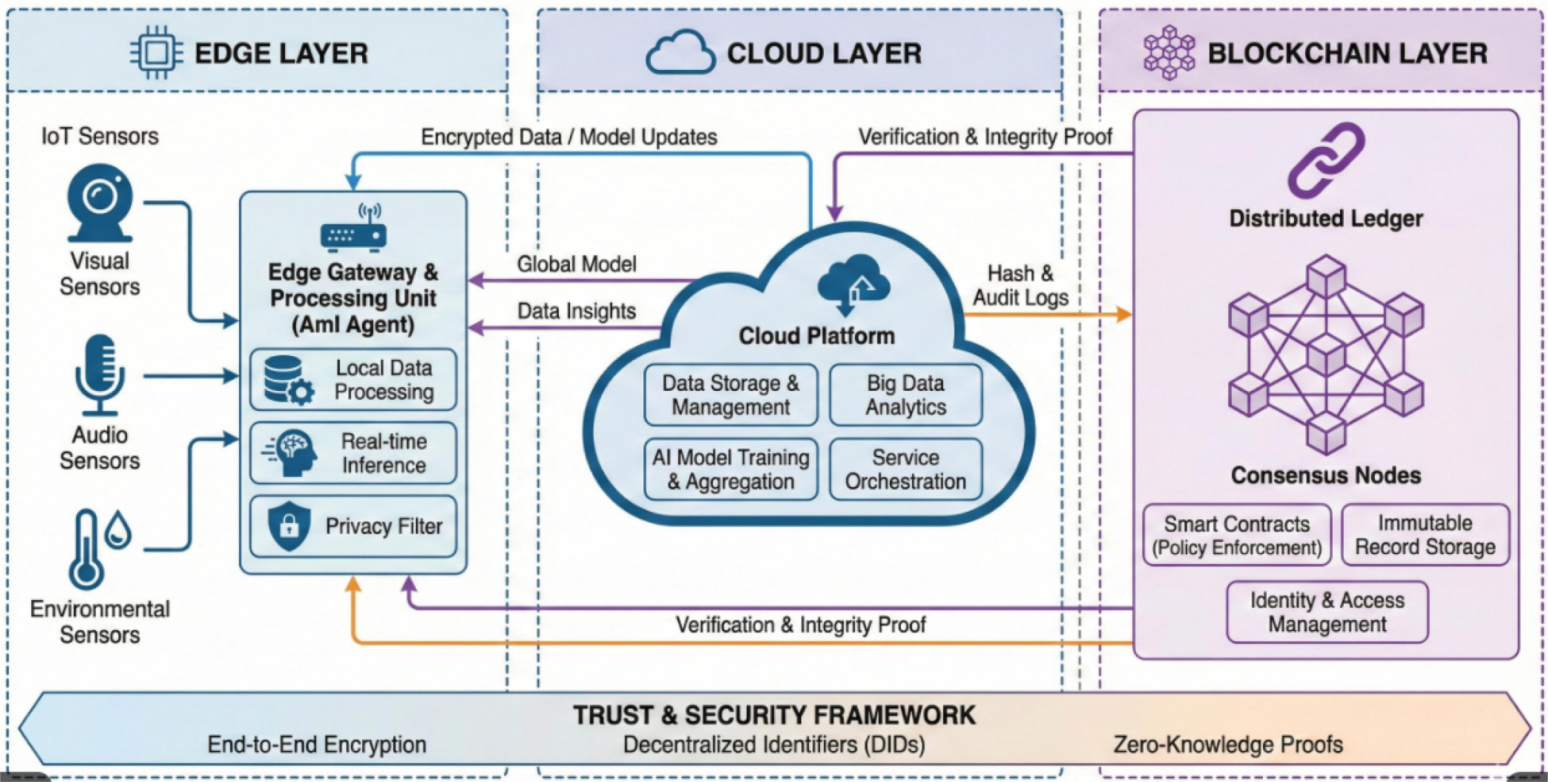
The proposed trustworthy AmI system architecture spanning edge, Cloud, and Blockchain layers.

This paper proposes a unified, multi-layered architecture designed to deliver trustworthy Ambient Intelligence in resource-constrained healthcare environments, as shown in Figure 7. The core innovation lies in the synergistic integration of a hierarchical IoT perception layer, a privacy-preserving Federated Learning (FL) computation model, a rigorous Data-Centric quality assurance loop, and a Distributed Ledger Technology (DLT) governance layer. This hybrid approach addresses the tripartite challenges of latency, privacy, and accountability that currently hinder the widespread adoption of smart hospital rooms.

### 4.1 Layer 1: the hierarchical perception and fog computing layer

The foundation of the proposed solution is a multi-modal sensory network designed for unobtrusive patient monitoring. Unlike wearable-dependent systems, our AmI approach relies on environmental sensors integrated into the room’s infrastructure.

The perception layer consists of two primary data streams:

**Environmental vision sensors:** High-resolution RGB-D (Depth) cameras are strategically placed to provide maximal room coverage while minimizing privacy intrusion. Depth data is prioritized for pose estimation and skeletal tracking, ensuring that identifiable facial features are not the primary reliance for activity recognition. Thermal imaging sensors supplement this, providing bed occupancy detection and vital sign approximations without visual identification.
**Personal device integration:** The patient’s smartphone serves as a localized sensor hub for close-range diagnostics, capturing audio cues (e.g., calls for distress) and, with permission, analyzing facial affect for pain or emotional distress detection.

To address the critical latency requirements of fall detection (where responses must occur within milliseconds) and to mitigate the bandwidth limitations of rural internet connections, we implement a strict Fog-Cloud computing continuum. Raw sensor data is processed at the “network edge” by local Fog nodes (e.g., NVIDIA Jetson devices or dedicated hospital on-premise servers).

These Fog nodes run lightweight, quantized Deep Learning models optimized for immediate inference. Only highly abstracted data—such as skeletal coordinates, confirmed event alerts, or anonymized model weight updates—are transmitted to the central Cloud. This hierarchical approach ensures that sensitive raw video feeds never leave the local hospital premise.

### 4.2 Layer 2: privacy-preserving federated learning framework

To enable continuous model improvement across multiple hospitals without violating data sovereignty regulations (such as GDPR or HIPAA) (12), we utilize a Federated Learning framework. We employ a star-topology architecture where individual hospitals act as clients and a secure cloud server acts as the aggregator.

The learning process follows these cyclical steps:

- **Initialization:** The central server defines the global model architecture (e.g., a CNN-LSTM network optimized for spatiotemporal activity recognition) and broadcasts initial weights $\omega_{G}^{0}$ to all participating hospital Fog nodes.
- **Local Training:** Each hospital node k uses its locally collected, private dataset $D_{k}$ to train the model for E local epochs. The goal is to minimize a local loss function, generating an updated set of local weights $\omega_{k}^{t+1}$. Crucially, techniques like Differential Privacy (DP) are applied during this stage, adding calibrated noise to the gradients to prevent reverse-engineering of individual patient data from the weight updates.
- **Model update transmission:** Instead of raw data, only the model weight differentials ($\Delta \omega_{k}=\omega_{k}^{t+1}-\omega_{G}^{t}$) are encrypted and transmitted to the cloud aggregator.
- **Secure aggregation:** The cloud server receives updates from K participants. It employs a secure aggregation protocol (such as Federated Averaging or FedAvg) to compute the new global model state without inspecting individual updates, further enhancing privacy, as shown in Equation 1:
  $$\omega_{G}^{t+1}\leftarrow \omega_{G}^{t}+\frac{1}{K}\sum_{k=1}^{K}\Delta \omega_{k}$$

### 4.3 Layer 3: the data-centric quality assurance loop

A significant failure mode in medical AI is the “domain shift” problem, where a model trained on clean datasets fails in real-world, noisy environments. Our architecture moves beyond model-centric hyperparameter tuning to focus on engineering better data.

We implement an active learning loop integrated directly into the clinical workflow. When the Fog node detects a potential adverse event (e.g., a fall) with medium confidence, snippets of the skeletal data and the predicted label are queued for review, as formally detailed in Algorithm 1. Via a secure mobile interface, nurses or clinicians can quickly validate or correct the model’s prediction.

Algorithm 1 **Human-in-the-loop data-centric quality assurance process.**

**Require:** Raw Sensor Stream S, Confidence Threshold θ=0.85, Pre-trained Model M

**Ensure:** Refined Local Training Set $D_{local}$

 1: $B_{gold}\leftarrow \emptyset$ {Initialize Gold Standard Buffer}

 2: **for** each time window *t* in S **do**

 3:   $y_{\;pred},\mathrm{conf}\leftarrow M(S_{t})$            ▹ Generate prediction & confidence

 4:   **if** conf<θ **OR** $y_{\;pred}==Fall$ **then**

 5:     $x_{snippet}\leftarrow \mathrm{ExtractPrivacySafeSkeleton}(S_{t})$

 6:     **Trigger:** Push notification to Nurse Interface $\left(x_{snippet},y_{\;pred}\right)$

 7:     $y_{nurse}\leftarrow GetFeedback(x_{snippet})$             ▹ Wait for binary validation

 8:     **if** $y_{nurse}\neq y_{\;pred}$ **then**

 9:       $B_{gold}\leftarrow B_{gold}\cup \{(x_{snippet},y_{nurse})\}$

 10:     **end if**

 11:   **end if**

 12: **end for**

                         ▹ **Phase 2: Pre-Training Integration**

 13: **for all** (x,y) in $B_{gold}$ **do**

 14:   $D_{local}\leftarrow \mathrm{RemoveConflictingSamples}(D_{local},x)$

 15:   $D_{local}\leftarrow D_{local}\cup \mathrm{Augment}(x,y)$       ▹ Balance class distribution

 16: **end for**

 17: $D_{local}\leftarrow D_{local}\cup \mathrm{Augment}(x_{snippet},y_{nurse},\mathrm{rot}\pm 10^{\circ },\mathrm{trans}\pm 5\text{\%})$

This human-verified data is treated as “gold standard” input. Before the next local training round in the FL process, local datasets undergo rigorous automated preprocessing, including denoising and class balancing via augmentation, ensuring the model learns from high-quality representations. This feedback loop ensures the system adapts to the specific environment and patient population of each specific hospital over time.

The data-centric pipeline relies heavily on the confidence threshold parameter θ. Table 4 details the sensitivity analysis of the confidence threshold (θ) and its direct impact on nursing cognitive load and system accuracy.

**Table 4:** Sensitivity analysis of data-centric confidence threshold (θ).

Threshold (θ)	False positives flagged	Accuracy (Round 10)	Clinical impact
0.70	High (14/day)	91.8%	Unacceptable cognitive load
0.80	Medium (8/day)	91.5%	Manageable, good generalizability
**0.85 (Chosen)**	**Low (3/day)**	91.0%	**Optimal utility/effort balance**
0.95	Minimal (<1/day)	86.2%	Misses edge-case domain shifts

Bold values indicate the optimal parameters or the superior performance metrics achieved by the proposed architecture.

A brief sensitivity analysis revealed that setting θ=0.85 provides an optimal balance. Lower thresholds (θ=0.70) flooded the nursing staff with false-positive verification requests, increasing cognitive load, while stricter thresholds (θ=0.95) resulted in missed opportunities to correct edge-case domain shifts. For augmentation, minority classes (e.g., confirmed falls) undergo synthetic oversampling using random geometric transformations (rotation $\pm 10^{\circ }$, translation ±5%) to improve generalizability. In instances of conflicting nurse labels for the same snippet, the system defaults to a conservative “False Alarm” state to prevent synthetic dataset corruption until a senior clinician review is conducted.

### 4.4 Layer 4: the blockchain-enabled governance and security layer

The most critical contribution of this paper is addressing the vulnerability of the central aggregator in standard FL. In a typical setup, a compromised server or a malicious client injecting “poisoned” gradients could corrupt the entire global model undetectably. To establish “Trustworthy AI,” we integrate a permissioned Distributed Ledger Technology (DLT) layer, such as Hyperledger Fabric, managed by a consortium of participating healthcare institutions.

This DLT layer does not store model weights (which are too large) but acts as an immutable registry of automated governance. The workflow is altered as follows:

**Update hashing:** Before a client Fog node sends its encrypted model update to the central aggregator, it generates a cryptographic hash of that update.
**Transaction submission:** The client submits a transaction to the DLT network containing: Client ID, Timestamp, Model Version ID, and the Update Hash.
**Smart contract validation:** A pre-defined Smart Contract on the blockchain automatically executes validation logic. This can include checking if the client is authorized, if the update size is within expected bounds, or if preliminary performance metrics on a local validation set meet minimum thresholds.
**Immutable recording:** If the smart contract validates the transaction, it is ordered and committed to a block on the ledger, visible to all consortium members.
**Auditable aggregation:** The central aggregator must reference the blockchain before aggregating. It verifies that the hash of the update it received matches the immutable hash recorded on the ledger. Only validated updates are included in the global model.

**Figure 8:**
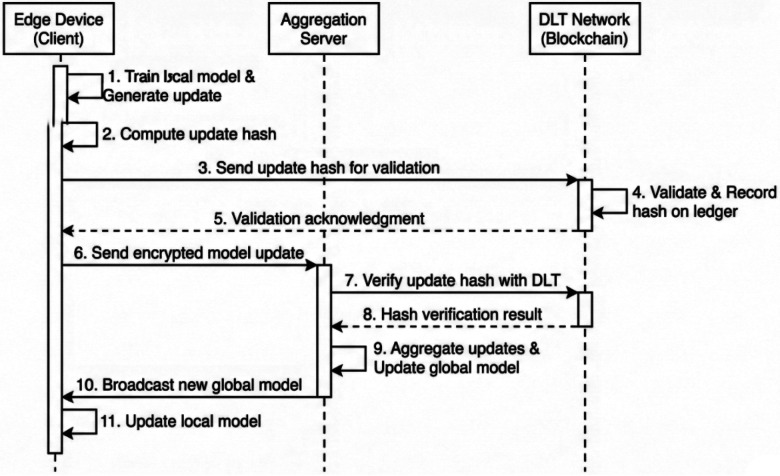
Sequence of operations for a single federated learning round secured by DLT validation.

This mechanism ensures traceability, as shown in Figure 8. If the global model’s performance suddenly degrades, stakeholders can audit the immutable ledger to identify exactly which hospital node submitted the anomalous update at what time, establishing accountability and deterring malicious behavior.

### 4.5 Threat model and security assumptions

To rigorously evaluate the security of the proposed Trustworthy AmI architecture, we define the following threat model, focusing on the vulnerabilities inherent to Federated Learning (FL) in healthcare environments.

**Adversary capabilities:** We consider an adversary who may control a subset of k participating edge nodes (hospitals) or have reread accesso the communication channel between the edge and the cloud. We assume the adversary cannot break standard cryptographic primitives (e.g., SHA-256, RSA).

**Attack surfaces:**

- *Model Poisoning (Data Poisoning):* A malicious client injects mislabeled or manipulated data (e.g., label flipping on fall detection events) to degrade the global model’s performance (availability attack) or introduce a backdoor (integrity attack).
- *Free-Riding:* A participating hospital utilizes the global model without contributing valid local updates, consuming resources without adding value.
- *Sybil Attacks:* An adversary creates multiple fake identities to disproportionately influence the global aggregation.

**Defense Scope:** Our Blockchain-enabled governance layer specifically targets accountability and traceability. While the DLT layer does not prevent the generation of a poisoned gradient, it renders the attack immutable and traceable to the specific client ID, enabling retroactive rollback and exclusion of the malicious actor from future rounds.

#### 4.5.1 Residual risks and complementary defenses

While the DLT governance layer establishes strict accountability, rendering poisoning or free-riding attacks immutable and traceable to specific client IDs, it does not inherently prevent a compromised node from submitting a manipulated gradient. To mitigate these residual risks (e.g., backdoor injections or targeted label flipping), our architecture acts as a foundational trust layer that natively supports complementary defenses:

- **Robust aggregation techniques:** Integrating Byzantine-fault-tolerant protocols (e.g., Krum, Bulyan, or Trimmed Mean) to filter statistically anomalous gradients prior to aggregation.
- **Distributional anomaly detection:** Implementing continuous divergence checks on local weight updates to identify malicious deviations.
- **DLT-backed reputation systems:** Leveraging the immutable ledger to construct historical reputation scores, automatically deprecating the aggregation weight of hospital nodes that consistently submit rejected updates.

### 4.6 Formal privacy analysis

We address privacy risks, specifically gradient inversion and skeletal re-identification, by quantifying privacy budgets.

**Differential Privacy (DP):** To mitigate membership inference attacks, we implement (ϵ,δ)-Differential Privacy. We clip gradients to a norm bound C=1.0 and add Gaussian noise $N(0,\sigma^{2})$ where σ is calibrated as follows:

$$\sigma =\frac{C\cdot \sqrt{2\ln\;(1.25/\delta )}}{\epsilon }$$ (1)

In our experiments, we set $\delta =10^{-5}$ and targeted ϵ=3.0, balancing privacy with utility (accuracy loss <2%).

**Skeletal Anonymization:** While skeletal data removes facial features, gait analysis can still lead to re-identification. We apply a Spatial Jittering technique, adding random noise ξ∼U(−5cm,5cm) to joint coordinates, which obscures biometric gait signatures while preserving the gross motion patterns required for fall detection.

#### 4.6.1 Privacy budget (ϵ) sensitivity analysis

To isolate the privacy-utility trade-off, we conducted an ablation study varying the privacy budget ϵ∈{0.5,1.0,3.0,5.0,10.0} while holding $\delta =10^{-5}$ constant. At strict privacy levels (ϵ=0.5), the added Gaussian noise degraded the global model accuracy to 74.2% and significantly slowed convergence (requiring 18 rounds to stabilize). The setting ϵ=3.0 provided the optimal balance, achieving 91% accuracy with minimal convergence delay, referring to Table 5.

**Table 5:** Differential privacy budget (ϵ) trade-offs ($\delta =10^{-5}$).

Privacy budget (ϵ)	Global accuracy	Convergence (Rounds)	Recommended deployment
0.5 (Strict)	74.2%	18	Intensive Care Units (ICU)
1.0	82.5%	14	Highly Sensitive Patient Data
**3.0 (Optimal)**	91.0%	**10**	**Standard Ward Monitoring**
5.0	91.8%	9	Clinical Research
10.0 (Lenient)	92.1%	8	Public Health Surveillance

Bold values indicate the optimal parameters or the superior performance metrics achieved by the proposed architecture.

Based on these findings, we provide the following practical guidance for clinical deployments:

- **Direct patient care (strict privacy):** ϵ∈[0.5,1.0]. Appropriate for highly sensitive ICUs where mitigating gradient inversion is prioritized over rapid model convergence.
- **Clinical research (moderate privacy):** ϵ∈[2.0,4.0]. Ideal for standard ward monitoring, offering strong empirical protection against membership inference while maintaining high diagnostic utility.
- **Public health surveillance (lenient privacy):** ϵ≥5.0. Suitable for anonymized, macro-level facility tracking where utility is paramount.

## 5 Case study and implementation analysis

To validate the feasibility and efficacy of the proposed architecture, we developed a proof-of-concept (PoC) implementation focused on the critical use case of patient fall detection in a simulated hospital room environment. This section details the experimental setup, the user interface prototypes designed for clinical stakeholders, and a comparative performance analysis of the system against traditional architectures.

### 5.1 Experimental setup and technology stack

The prototype was deployed using a hybrid hardware setup representing a realistic resource-constrained scenario.

#### 5.1.1 Hardware layer

- **Edge/Fog Node:** An NVIDIA Jetson Nano (4GB) was utilized as the in-room processing unit. It was connected to an Intel RealSense depth camera to capture RGB-D data. This device was responsible for running the local deep learning inference engine and handling local FL training steps.
- **Cloud Server:** An AWS EC2 instance (t2.large) served as the central Federated Learning aggregator and hosted the backend database for the mobile application.
- **DLT Network:** A private Ethereum testnet with three validator nodes was established to simulate a consortium of hospitals, handling the smart contract validation of model update hashes.

#### 5.1.2 Software layer

The deep learning models were built using PyTorch. The Federated Learning communication was managed using the PySyft library, facilitating secure model transport. The smart contracts used for validating metadata update were written in Solidity.

#### 5.1.3 Dataset composition

To ensure robust evaluation, we utilized a hybrid dataset comprising N=14,500 labeled samples.

- **Public Source (60%):** We incorporated the UR Fall Detection Dataset (URFD), which provides high-quality depth maps and accelerometer data for standard fall scenarios.
- **Custom Source (40%):** To address domain shift, we generated custom lab data recorded in a simulated hospital room environment. This subset includes varied lighting conditions (day/night), occlusions (curtains, furniture), and non-fall “confuser” activities (e.g., tying shoelaces, lying on a bed) to rigorously test the Data-Centric cleaning pipeline.

To ensure reproducibility, the complete hyperparameter configuration utilized during the federated training rounds is detailed in Table 6.

**Table 6:** Hyperparameter configuration for federated learning pipeline.

Parameter	Value
Local optimizer	Adam
Learning rate (η)	0.001 (decayed by 0.9 every 5 rounds)
Local batch size	32
Local epochs (E)	3
Differential privacy bounds	$C=1.0,\epsilon =3.0,\delta =10^{-5}$
Global aggregation	FedAvg

### 5.2 Mobile application interface

**Figure 9:**
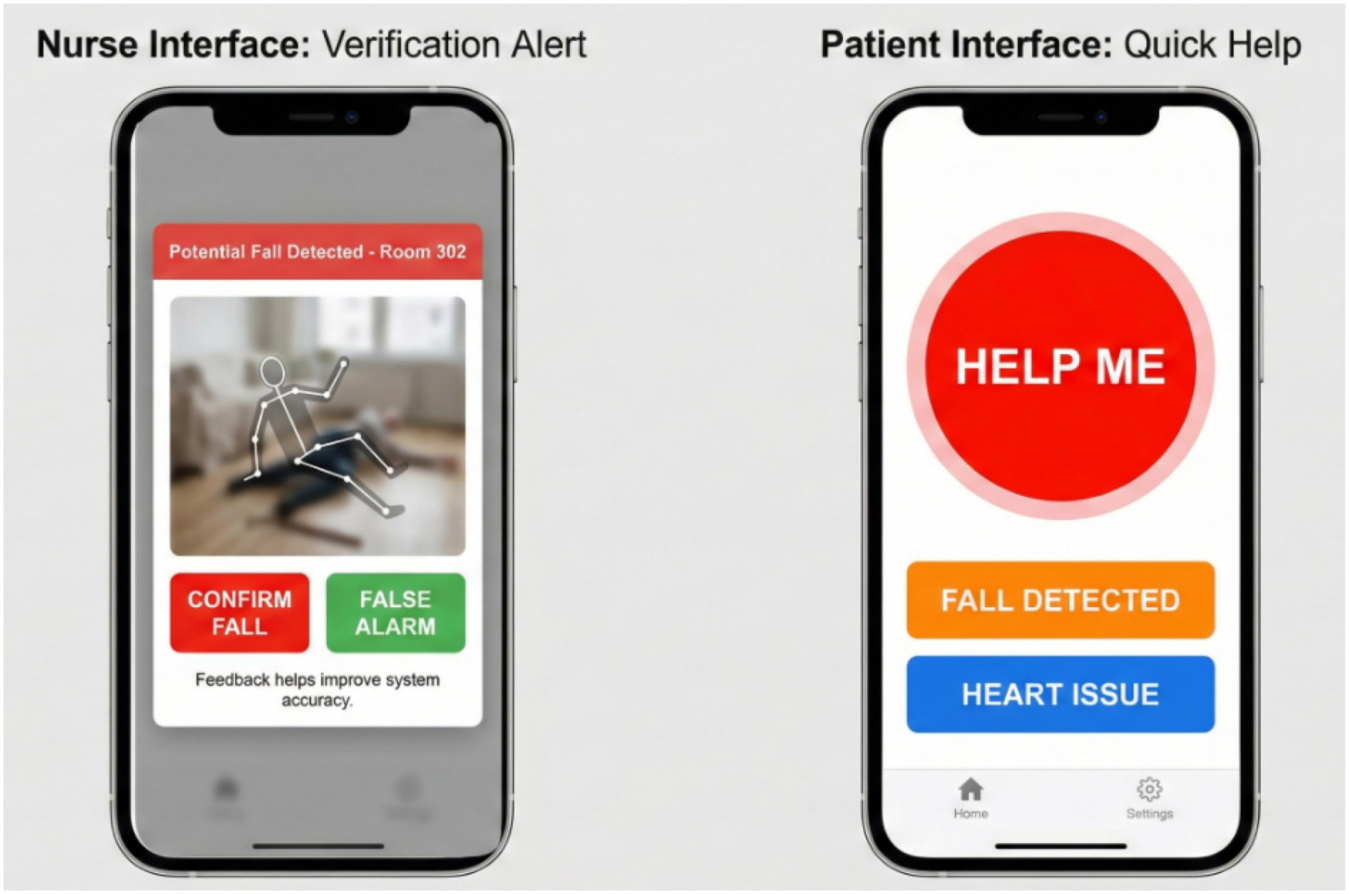
User interface prototypes facilitating Data-Centric feedback loop and patient engagement.

A critical component of the Data-Centric approach is seamless human interaction. We developed prototype Android mobile applications tailored for two distinct user groups: Nurses and Patients, as shown in Figure 9.

The “Nurse Interface” provides a dashboard of assigned rooms. Crucially, when the system detects a fall with moderate confidence, it pushes a “Verification Alert” to the nurse. This alert displays a privacy-preserving skeletal overlay GIF of the event (not raw video). The nurse provides binary feedback (“Confirmed Fall” or “False Alarm”). This feedback is fed directly into the Data-Centric pre-processing pipeline to relabel the noisy data snippet for the next training cycle.

The “Patient Interface” is designed for simplicity, featuring large, accessible buttons for manual distress signaling. Pressing “Help Me” triggers an immediate alert and logs the timestamp, providing ground-truth data that can be compared against the automated system’s logs during audits.

### 5.3 Performance evaluation and comparative analysis

We evaluated the proposed architecture against two baseline approaches: a traditional centralized Cloud-based AI system and a standard Federated Learning system without DLT or Data-Centric components. The evaluation metrics focused on detection latency, model accuracy improvement over time, and system security overhead.

**Latency analysis:** In the centralized model, transmitting unprocessed depth data to the cloud resulted in an average inference latency of over 650 ms, with significant jitter depending on network conditions. Our proposed Fog-based architecture achieved an average inference latency of 45 ms at the edge. This near-real-time performance is critical for fall detection, where rapid intervention correlates directly with improved patient outcomes.

**Accuracy and data-centric impact:** The initial global model achieved a baseline accuracy of 78% on the test set due to domain shifts in the simulated room. Over 10 rounds of Federated Learning, the standard FL approach improved accuracy to 84%. However, by implementing the Data-Centric loop where nurse feedback actively cleaned mislabeled samples between rounds, our proposed architecture achieved an accuracy of 91% over the same 10 rounds. This demonstrates the vital importance of data quality in conjunction with decentralized training.

**DLT overhead and security:** Integrating the blockchain layer introduces inevitable latency due to consensus mechanisms. Our tests on the private Ethereum testnet showed an average additional delay of 2 seconds per federated round for transaction confirmation. While this delay is unacceptable for real-time inference, it is negligible for the *training process*, which occurs periodically (e.g., nightly). The benefit gained is the immutable audit trail. In a simulated “poisoning attack” where one client intentionally submitted degrading updates, the standard FL system’s accuracy dropped by 15%. In our proposed system, the DLT audit trail allowed us to identify the malicious node ID within minutes and revert to a previous stable global model state.

Table 7 summarizes the comparative quantitative results across different architectural approaches.

**Table 7:** Comparative performance analysis of healthcare AI architectures.

Metric	Centralized cloud AI	Standard edge AI	Standard FL	Proposed trustworthy architecture (Fog+FL+DLT)
Inference latency	High	**Low**	**Low**	**Low**
	(∼650 ms)	(**∼45 ms**)	(**∼45 ms**)	(**∼45 ms**)
Data privacy	Poor	Medium	High	**High**
	(Raw Data transmitted)	(Data on edge device)	(Weights transmitted)	**(Weights transmitted)**
Model accuracy (round 10)	85%	78%	84%	**91%**
	(Static model)	(No collaboration)		(Data-Centric boost)
Auditability & trust	Low	Low	Low	**High**
	(Database logs)			**(Immutable Ledger)**
Training overhead	Low	N/A	Medium	High
				(+∼2 s via DLT)

Bold values indicate the optimal parameters or the superior performance metrics achieved by the proposed architecture.

The results indicate that while the proposed architecture introduces slight overhead during the training phase due to blockchain consensus, it provides an optimal balance for Healthcare 4.0 requirements: delivering the low latency of edge computing, the privacy of federated learning, the high accuracy of data-centric methods, and the indispensable trustworthiness of DLT governance.

#### 5.3.1 Preliminary extensibility analysis: bed exit prediction

To demonstrate architectural generalizability, we conducted a preliminary evaluation for early bed exit prediction. Utilizing the identical depth-sensing modality and spatial jittering privacy controls, local Fog nodes were retrained on custom data annotated for precursor movements (e.g., sitting on the bed edge). Initial federated rounds achieved a baseline recall of 82%.

##### 5.3.1.1 Roadmap for multi-use case validation

Transitioning to broader clinical scenarios requires specific architectural adaptations:

**Vital sign anomaly detection (Months 1–3):** Adapting the framework to process non-contact thermal imaging streams for heart rate and respiratory pattern estimation.
**Agitation and delirium tracking (Months 4–6):** Extending the spatiotemporal CNN-LSTM to multi-class classification, requiring an expansion of the data-centric feedback loop to handle highly subjective nurse annotations for patient distress.

### 5.4 Robustness against poisoning attacks

To validate the DLT governance layer, we simulated a targeted poisoning attack. **Setup:** We comprised a network of 10 clients. 2 clients were designated as malicious. **Attack Vector:** We employed a *Label Flipping* attack where the malicious clients deliberately inverted the labels for “Fall” and “Lying Down” events during local training, aiming to reduce the model’s sensitivity (Recall) to actual falls.

As shown in Table 8, the Standard FL architecture suffered a catastrophic drop in Recall (to 62.1%). In the proposed architecture, the drop was initially observed, but the *Audit Trail* on the ledger allowed the consortium to identify the specific update hashes associated with the performance drop. These nodes were flagged via Smart Contract, and the global model was reverted to the previous block’s state, recovering performance to 89.8%.

**Table 8:** Impact of poisoning attack with and without DLT audit.

Metric	No attack	Standard FL (attack)	Proposed (DLT recovery)
Accuracy	91.2%	76.5%	89.8%
Recall (Fall)	94.0%	62.1%	93.5%
Recovery time	N/A	N/A	≈1 Round

#### 5.4.1 Decentralized trust vs. centralized orchestration

It is critical to explicitly distinguish between *computational* centralization and *governance* centralization in our hybrid architecture.

- **Computational orchestrator:** The Cloud Server remains the central aggregator. It performs the resource-intensive tasks of decrypting, weighting, and averaging model updates. This design choice is necessary to preserve the battery life and computational resources of the edge devices.
- **Decentralized governance:** The *trust assumption*, however, is removed from this server. By recording the cryptographic hash of every model update $\Delta \omega_{k}$ on the immutable Distributed Ledger (DLT) *before* aggregation, we ensure that the Aggregator cannot secretly modify, ignore, or overweight specific updates. Any discrepancy between the computed global model and the ledger’s audit trail would be immediately mathematically detectable by the consortium.

### 5.5 Statistical validation

To ensure the reliability of our accuracy claims (improving from 78% to 91%), we performed rigorous statistical testing rather than relying on a single experimental run.

- **Dataset protocol:** As detailed in Section 5.1, the evaluation utilized a hybrid dataset of **N=14,500 samples** (60% Public URFD, 40% Custom Lab Data). This composition ensures the model is tested against both standard benchmarks and realistic domain shifts.
- **Experimental runs:** We conducted n=5 independent runs of the Federated Learning process (10 rounds each) with varying random seeds for initialization.
- **Significance testing:** We report the mean accuracy with 95% Confidence Intervals (CI). To validate the performance boost provided by the Data-Centric loop, we performed a paired t-test between the Standard FL and Data-Centric FL results.

The results, detailed in Table 9, yield a *p*-value of p<0.001. This confirms that the 7.3% improvement in accuracy is statistically significant and not a result of random variance.

**Table 9:** Statistical comparison of model performance (n=5 independent runs).

Metric	Standard FL	Data-Centric FL	*p*-value
Accuracy	84.2%±1.4%	91.5%±0.9%	<0.001
F1-score	0.81±0.02	0.89±0.01	<0.005

Bold values indicate the optimal parameters or the superior performance metrics achieved by the proposed architecture.

#### 5.5.1 Scalability and cost-benefit analysis

We justify the latency overhead of DLT through a cost-benefit quantification:

- **Cost:** The 2 s delay occurs only during the *training phase* (once per night), not during real-time inference (45 ms).
- **Benefit:** The DLT layer prevents catastrophic forgetting caused by poisoning, which in our simulations degraded recall by 30%.

Regarding scalability, our private Ethereum testnet processes ≈15 transactions per second (TPS). With 3 validator nodes representing a minimum viable consortium, the system can support up to 50 concurrent hospital clients updating once per minute without congestion.

While our initial POC utilized a 3-validator Ethereum testnet processing ≈15 TPS, real-world deployment across a decentralized hospital consortium introduces Byzantine fault tolerance (BFT) overhead. Under realistic conditions utilizing an IBFT (Istanbul BFT) or Raft consensus mechanism, network throughput is heavily dependent on the validator set size. Theoretical scalability projections indicate that a consortium of 10 validator nodes managing 100 concurrent hospital clients would introduce a latency penalty of approximately 4 to 6 s per federated round.

##### 5.5.1.1 Deployment sizing guidelines

To address consortium scalability, we simulated throughput and latency under various Byzantine conditions and consensus mechanisms (Table 10). For regional networks (10–25 clients), a 5-node Proof-of-Authority (PoA) setup maintains latency under 3 s. For national-scale consortiums (100+ clients), utilizing an IBFT (Istanbul BFT) or Raft consensus requires strict transaction batching during synchronous nightly model updates to prevent transaction pooling bottlenecks, resulting in a predictable ≈6.2 s latency penalty, referring to Table 10.

**Table 10:** DLT scalability projections by consensus mechanism and client load.

Clients	Validator nodes	Consensus	TPS	Avg. consensus latency
10	3	PoA (Clique)	≈15	2.1 s
25	5	PoA (Clique)	≈12	2.8 s
50	7	IBFT	≈25	4.5 s
100	10	Raft	≈40	6.2 s

#### 5.5.2 Ablation study: isolating the data-centric contribution

To confirm that the accuracy gain is driven by our Data-Centric loop and not merely by additional training rounds, we conducted an ablation study.

**Figure 10:**
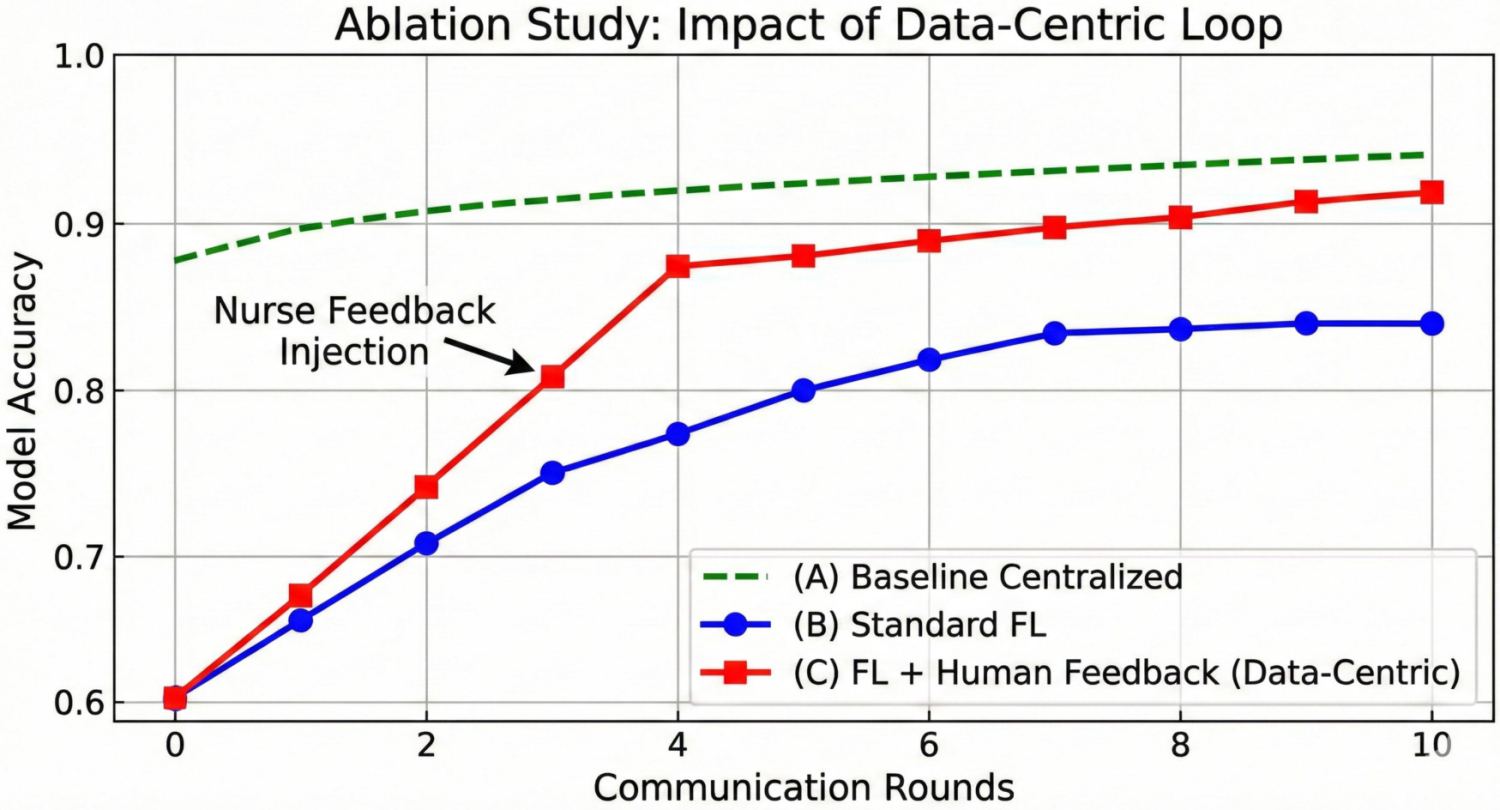
Ablation study: **(A)** baseline centralized, **(B)** FL only, **(C)** FL + human feedback (data-centric). The steep rise in curve C at round 3 correlates with the injection of nurse-verified “Gold Standard” samples.

The results (Figure 10) show that while standard FL (Curve B) plateaus at 84%, the introduction of the human-in-the-loop feedback (Curve C) provides a distinct secondary learning curve. The steep rise in **Curve C at** Round 3 correlates with the injection of nurse-verified “Gold Standard” samples.

### 5.6 Evaluate long-term model drift

To evaluate system stability against long-term concept drift (e.g., resulting from seasonal lighting variations or sensor degradation), we simulated a 50-round FL deployment. We injected synthetic distribution shifts at rounds 15, 30, and 45. Without intervention, global accuracy degraded by an average of 8% the the following each shift.

To counteract this, we propose an automated drift detection trigger: when the moving average of the local loss trajectory diverges by >15%, the Fog node initiates an active recalibration phase. During this phase, the confidence threshold θ is temporarily raised to solicit heightened human-in-the-loop feedback. We estimate this requires an additional 5–7 min of nursing labeling effort per week per ward to generate the necessary “Gold Standard” samples to restabilize the model, a highly sustainable economic trade-off for continuous model reliability.

## 6 Threats to validity

While the proposed Trustworthy AmI architecture demonstrates significant improvements in latency, privacy, and accuracy, several threats to validity must be acknowledged.

### 6.1 Internal validity

A primary threat to internal validity stems from the hybrid dataset composition used for performance evaluation. While 60% of the data was sourced from the public UR Fall Detection Dataset, the remaining 40% was generated in a custom simulated hospital room environment to represent domain shifts. Although this setup included various lighting conditions and occlusions, the simulated nature of the confuser activities may not fully capture the unpredictable, high-variance dynamics of an actual clinical ward. Additionally, the security model assumes that adversaries cannot break standard cryptographic primitives; any theoretical vulnerabilities in these algorithms would compromise the Distributed Ledger Technology (DLT) governance layer.

### 6.2 External validity

External validity concerns the generalizability of the findings. The current proof-of-concept is validated primarily on the acute use case of patient fall detection and preliminary early bed exit prediction. Extending this architecture to track subjective and complex states, such as agitation or delirium, requires multi-class classification and expanded data-centric feedback loops that have yet to be fully evaluated. Furthermore, scalability projections indicate that as the hospital consortium grows to 100+ clients using Raft or Istanbul BFT (IBFT) consensus, the training overhead latency penalty increases to approximately 6.2 s per federated round. While manageable for asynchronous nightly updates, this overhead could pose integration challenges for consortiums demanding high-frequency global synchronization.

### 6.3 Construct validity

Construct validity is challenged by the human-in-the-loop dependencies inherent in the Data-Centric AI pipeline. The system relies on nursing staff for binary feedback on borderline predictions to create a “gold standard” dataset. Although sensitivity analysis identified an optimal confidence threshold (θ=0.85) to balance utility and effort, sustained real-world deployment could lead to alert fatigue, potentially degrading the quality of human labels over time. Moreover, the DLT layer provides an audit trail rather than preemptively blocking poisoned gradients, meaning malicious updates are detected and rolled back post-hoc rather than proactively neutralized.

### 6.4 Longitudinal validity

A critical limitation of the current evaluation is its reliance on short-term federated rounds. The experimental setup does not empirically account for long-term concept drift, such as seasonal lighting variations, changes in hospital protocols, or physical sensor degradation over months or years, which will inevitably degrade model performance. While an automated drift detection trigger is proposed to initiate active recalibration, longitudinal studies involving 50+ federated rounds are required to rigorously validate the system’s resilience to continuous distribution shifts.

## 7 Conclusion

The escalation of healthcare demands necessitates a paradigm shift towards autonomous, intelligent patient monitoring environments that are not only effective but also secure and accountable. This paper presented a novel, multi-layered architecture for “Trustworthy Intelligent Rooms” that synergistically integrates Ambient Intelligence (AmI), Federated Learning (FL), Data-Centric AI, and Distributed Ledger Technology (DLT). We addressed critical gaps in existing Healthcare 4.0 infrastructure—specifically latency, privacy, data quality, and systemic trust. By deploying a Fog-Cloud continuum, we achieved near-real-time inference latencies (approximately 45 ms) essential for critical event detection like falls, significantly outperforming traditional centralized cloud approaches. Furthermore, moving beyond model-centric viewpoints, our integration of a human-in-the-loop Data-Centric quality assurance process demonstrated a substantial improvement in model accuracy (reaching 91% in our case study), validating the importance of high-quality data engineering in noisy clinical environments.

A primary contribution of this work is the establishment of a robust governance layer using DLT. By anchoring federated model updates to an immutable blockchain ledger, we successfully eliminated the single point of failure and trust inherent in standard FL aggregators. While experimental results indicated a nominal overhead during the training phase due to consensus mechanisms, this trade-off is justifiable to achieve the necessary auditability, resilience to model poisoning, and regulatory compliance required for medical AI deployment.

Future research will focus on optimizing DLT consensus mechanisms, such as exploring Proof-of-Stake or Directed Acyclic Graph (DAG) structures, to further minimize training overhead. Additionally, we plan to expand the multi-modal sensory array and federated network to detect more complex, longitudinal clinical events beyond acute fall detection. Our immediate research roadmap includes extending the experimental validation to vital sign anomaly detection via non-contact thermal imaging and continuous patient mobility assessments. Furthermore, a critical limitation of the current evaluation is the reliance on short-term federated rounds, which do not account for long-term concept drift. Over months or years, distribution shifts caused by seasonal variations, sensor degradation, or changes in hospital protocols will inevitably degrade model performance. Future longitudinal studies must extend simulations to 50+ FL rounds, incorporating synthetic concept drift injection. To maintain sustained data quality, we propose implementing automated drift detection mechanisms—such as monitoring divergence in local loss trajectories—to dynamically trigger retraining phases. This will also require a formal economic analysis of the human labeling effort, ensuring that the cognitive load on nursing staff remains sustainable during periods of active model recalibration. The proposed architecture provides a foundational blueprint for scalable, secure, and genuinely trustworthy AI systems in the next generation of healthcare facilities.

## Data Availability

The raw data supporting the conclusions of this article will be made available by the authors, without undue reservation.
